# Disruption of mechanical stress in extracellular matrix is related to Stanford type A aortic dissection through down-regulation of Yes-associated protein

**DOI:** 10.18632/aging.101033

**Published:** 2016-09-05

**Authors:** Wen-Jian Jiang, Wei-Hong Ren, Xu-Jie Liu, Yan Liu, Fu-Jian Wu, Li-Zhong Sun, Feng Lan, Jie Du, Hong-Jia Zhang

**Affiliations:** ^1^ Department of Cardiac Surgery, Beijing Anzhen Hospital, Capital Medical University, Beijing 10029, China; ^2^ Beijing Institute of Heart, Lung and Blood Vessel Diseases, Beijing 10029, China; ^3^ Beijing Laboratory for Cardiovascular Precision Medicine, Beijing, China, 10029; ^4^ The Key Laboratory of Remodeling-related Cardiovascular Disease, Ministry of Education, Beijing 10029, China; ^5^ Beijing Aortic Disease Center, Cardiovascular Surgery Center, Beijing 10029, China; ^6^ Beijing Engineering Research Center for Vascular Prostheses, Beijing 10029, China

**Keywords:** extracellular matrix, mechanical stress, yes-associated protein, vascular smooth muscle cell, Stanford type A aortic dissection

## Abstract

In this study, we assessed whether the down-regulation of Yes-associated protein (YAP) is involved in the pathogenesis of extracellular matrix (ECM) mechanical stress-induced Stanford type A aortic dissection (STAAD). Human aortic samples were obtained from heart transplantation donors as normal controls and from STAAD patients undergoing surgical replacement of the ascending aorta. Decreased maximum aortic wall velocity, ECM disorders, increased VSMC apoptosis, and YAP down-regulation were identified in STAAD samples. In a mouse model of STAAD, YAP was down-regulated over time during the development of ECM damage, and increased VSMC apoptosis was also observed. YAP knockdown induced VSMC apoptosis under static conditions *in vitro*, and the change in mechanical stress induced YAP down-regulation and VSMC apoptosis. This study provides evidence that YAP down-regulation caused by the disruption of mechanical stress is associated with the development of STAAD via the induction of apoptosis in aortic VSMCs. As STAAD is among the most elusive and life-threatening vascular diseases, better understanding of the molecular pathogenesis of STAAD is critical to improve clinical outcome.

## INTRODUCTION

Aortic dissection (AD) is among the most elusive and life-threatening vascular diseases. When the ascending aorta is involved, the dissection is termed a Stanford type A aortic dissection (STAAD) [1]. In the natural history of aortic dissection, the mortality of untreated patients with acute STAAD is 32% in the first 24 hours, 50% at the end of the third day, and up to 80% two weeks after onset [2]. The molecular pathogenesis of STAAD is not yet well understood.

The ascending aortic wall consists of collagen, vascular smooth muscle cell (VSMC), and approximately 50 elastic laminas [3]. Previous studies have shown that histopathological and genetic factors lead to reduced elasticity and media degeneration of the aortic wall, which disrupt the homeostasis of extracellular matrix (ECM) mechanical stress [4, 5]. Thus, pre-existing weaknesses of the aorta are considered the basis for aortic dissection [6, 7]. Mature VSMCs play a role in contractile function during homoeostasis. VSMC apoptosis has been implicated as a major event in many aortic diseases [8-10] and especially in the pathogenesis of aortic dissection [11]. VSMC functioning is also associated with changes in mechanical stress [12, 13]. However, how disorganized mechanical stress contributes to VSMC apoptosis and the development of STAAD remains unclear.

Yes-associated protein (YAP) is ubiquitous *in vivo* and involved in the regulation of cell proliferation and apoptosis [14-16]. Increased YAP expression and activation result in the initiation of proliferation and the suppression of apoptosis in hepatocellular carcinoma [17-20] and pancreatic progenitor cells [21]. Our previous study found that enlargement of cardio-myocytes, which is induced by YAP up-regulation, led to cardiac hypertrophy [22]. Recently, the ablation of smooth muscle–specific *Yap* in mice resulted in embryonic lethality with abnormal aorta development [23]. Thus, the functional role of YAP in cardiac/SMC proliferation during cardiovascular development cannot be overemphasized [23]. Moreover, altered mechanical stress reportedly affects YAP expression in tumor tissues [24, 25].

In this study, we investigated the relationship between YAP down-regulation and VSMC apoptosis during the development of STAAD. Furthermore, in a BAPN (β-aminopropionitrile monofumarate)-induced mouse STAAD model, we found that the changes in YAP expression observed in VSMCs are similar to those observed in clinical samples.

## RESULTS

### Maximum aortic wall velocity was decreased in STAAD ascending aorta

**Figure 1:**
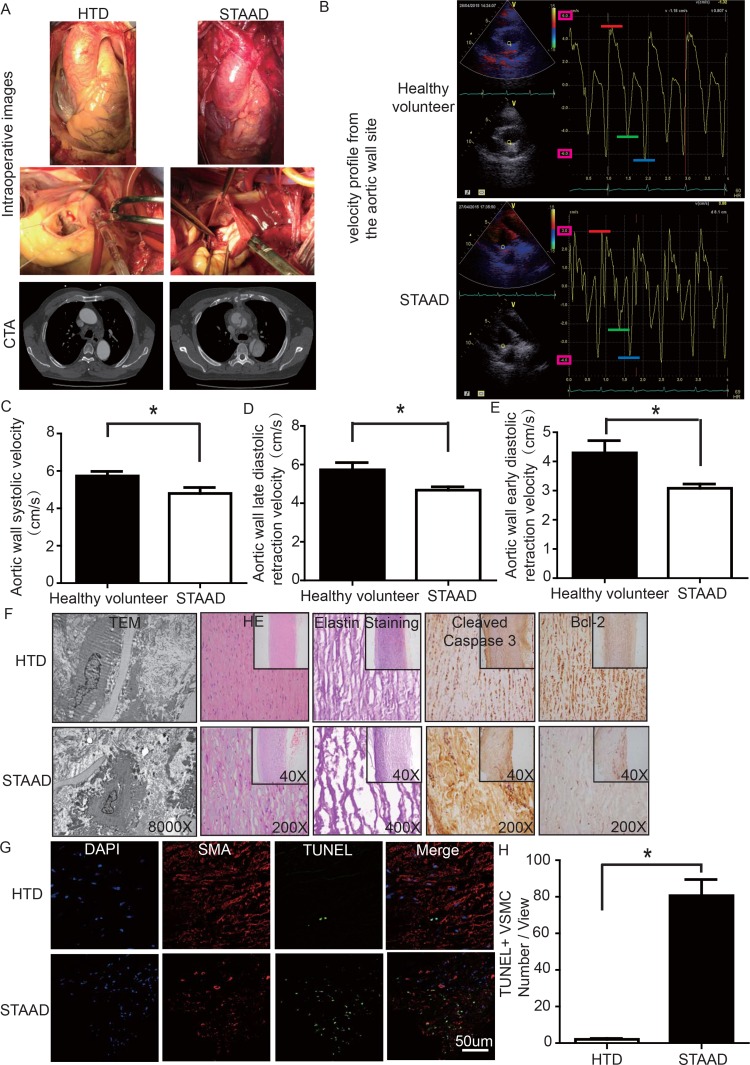
(**A**) Intraoperative images and CTA results showing the enlarged ascending aorta and typical true and false cavities in STAAD. (**B**) Echocardiography showing the Vmax of healthy volunteers and patients with STAAD (including aortic wall systolic velocity (red line), late diastolic retraction velocity (blue line) and early diastolic retraction velocity (green line)). (**C**) The mean aortic wall systolic velocity of the ascending aorta was significantly lower in patients with STAAD than in healthy volunteers (n=5 in healthy volunteer group, n=5 in STAAD group, *p=0.0417). (**D**) The mean late-diastolic retraction velocity of the ascending aorta was significantly lower in patients with STAAD than in healthy volunteers (n=5 in Healthy volunteer group, n=5 in STAAD group, *p=0.0478). (**E**) The mean early-diastolic retraction velocity of the ascending aorta was significantly lower in patients with STAAD than in healthy volunteers (n=5 in healthy volunteer group, n=5 in STAAD group, *p=0.0407). (**F)** TEM showed partly fragmented and reduplicated elastic lamina and abnormal VSMCs, together with an electron-dense amorphous material peripheral cell membrane in the ascending aortic wall of patients with STAAD, H&E and elastin staining showed obvious ascending aorta tissue structure disorganization in patients with STAAD, and immuno-histochemistry showed that cleaved caspase-3 was present at high levels and bcl-2 was present at low levels in the ascending aortic wall of patients with STAAD relative to that of HTDs. (**G**, **H**) Confocal fluorescence microscopy showed that the numbers of double stained (TUNEL and α-SMA) cells was higher in the ascending aortic wall of patients with STAAD (n=19 in HTD group, n=23 in STAAD group, *p=0.0009).

To determine whether there was a reduction of aortic wall elasticity, we collected samples from STAAD patients undergoing ascending aorta replacement and from heart transplantation donors (HTD). Representa-tive computed tomography and intraoperative images of selected STAAD patients and HTDs illustrating typical STAAD features are presented in Figure 1A. The ascending aortas of STAAD patients were demonstrably enlarged according to the intraoperative images and computed tomography angiography (CTA) (Figure 1A); typical true and false cavities were observed using CTA (Figure 1A). We also compared the STAAD patients to age- and gender-matched healthy volunteers: echocardiography revealed highly significant maximum aortic wall velocity (Vmax) differences (including aortic wall systolic velocity, late diastolic retraction velocity and early diastolic retraction velocity) between healthy subjects (Figure 1B, C-E) and patients with STAAD (Figure 1B, C-E). The mean aortic wall systolic velocity of the ascending aorta was significantly lower (*p*=0.0417) in patients with STAAD (4.80±0.32 cm/s) than that in healthy volunteers (5.73±0.25 cm/s). The mean late- and early-diastolic retraction velocities of the ascending aorta were 5.73±0.37 cm/s and 4.30±0.42 cm/s, respectively, in healthy volunteers and 4.68±0.17 cm/s and 3.08±0.15 cm/s, respectively, in patients with STAAD (late, *p*=0.0478; early, *p*=0.0407).

### The ascending aortic walls of patients with STAAD presented ECM disorders and increased VSMC apoptosis

We used histology to determine whether there were ECM disorders and increased VSMC apoptosis. H&E and elastin staining showed obvious VSMC disorganization and elastic lamellae dissection in the STAAD samples (Figure 1F). Relative to VSMCs in HTDs, the arrangement of the VSMCs in STAAD samples was sparse and irregular, the number of elastic lamellae decreased and the gaps among elastic lamellae were larger (Figure 1F). transmission electron microscope (TEM) analysis of human ascending aortic wall samples obtained from patients with STAAD showed obvious abnormalities compared to the normal control samples (Figure 1F). The aortic tunica media of the normal ascending aorta presented a regular appearance, and smooth muscle cells were observed between the elastic lamina within a homogenous interstitial matrix. The STAAD samples exhibited severe alterations in the vascular wall structure. The aortic media of STAAD showed irregular VSMCs between partly fragmented and reduplicated elastic lamina. In the STAAD samples, abnormal VSMCs were frequently observed together with an electron-dense amorphous material in the peripheral cell membrane; this finding is consistent with apoptosis (Figure 1F). Moreover, we also detected the VSMC apoptosis in the STAAD samples by confocal fluorescence microscopy analysis of terminal deoxynucleotidyl transferase-mediated nick-end labeling (TUNEL) and α-SMA staining (Figure 1G, H), as well as cleaved caspase-3 and Bcl-2 expression (Figure 1F). The results obtained were consistent with those obtained using TEM.

### YAP expression decreased in ascending aortic wall in patients with STAAD

**Figure 2:**
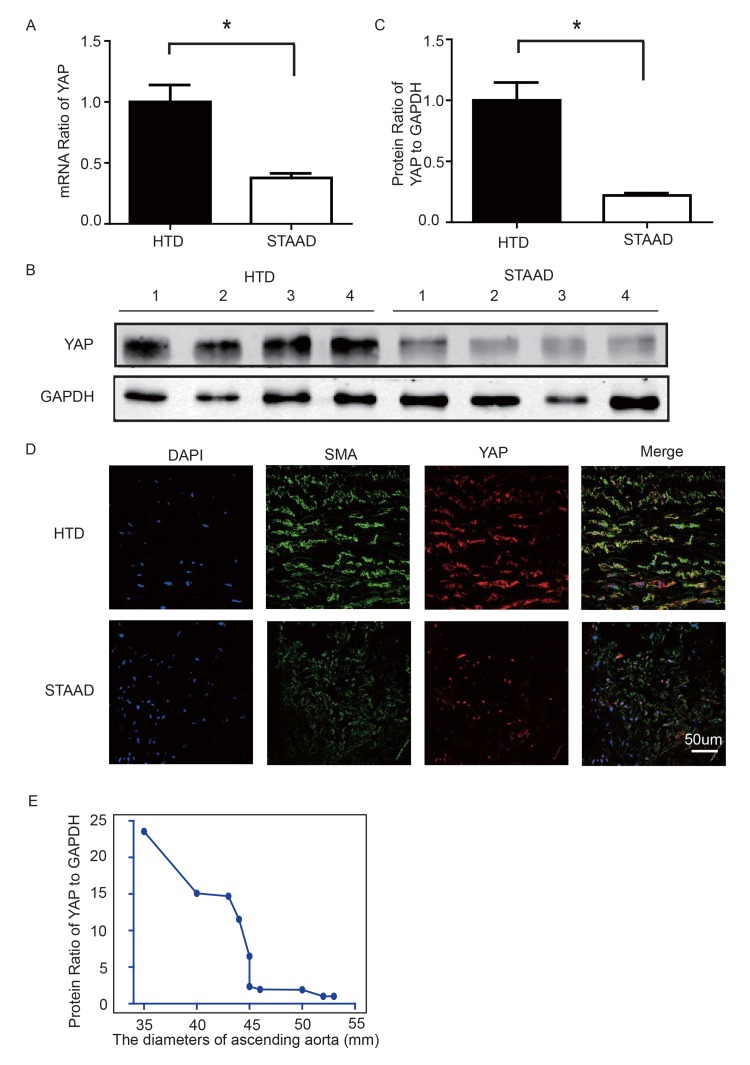
(**A**) Real-time PCR results showing that YAP was significantly down-regulated in the ascending aortic wall of patients with STAAD compared to HTDs (n=19 in HTD group, n=23 in STAAD group, *p=0.0003). (**B**, **C**) Western blotting showed that the expression of total YAP proteins was significantly lower in the ascending aortic wall of patients with STAAD compared to HTDs (n=19 in HTD group, n=23 in STAAD group, *p=0.0019). (**D**) Confocal fluorescence microscopy showing that YAP and α-SMA double stained cells were present at lower numbers and less quantity in patients with STAAD compared to HTDs. (**E**) Quantified and statistical analyzed of each patient sample's Western blotting result showed that YAP expression was negatively correlated with the ascending aorta diameter (n=10).

To observe YAP expression, we obtained quantitative data using western blotting and real-time PCR. We observed significant decreases in YAP RNA and protein expression in the ascending aortic wall of patients with STAAD (Figure 2A-C). Confocal fluorescence microscopy analysis of the co-staining of α-SMA and YAP in the tunica media indicated that YAP is mainly expressed in the VSMCs in normal aortas; in STAAD, the expression level of YAP in the middle layer is reduced (Figure 2D). Moreover, SMA expression was lower in STAAD, demonstrating that VSMCs were lost (Figure 2D). Additionally, YAP expression was negatively correlated with the ascending aorta diameter (Figure 2E), indicating that YAP may be involved in STAAD development.

### BAPN-induced mouse STAAD model recapitulated human phenotype, showing damaged ECM and VSMC apoptosis

**Figure 3:**
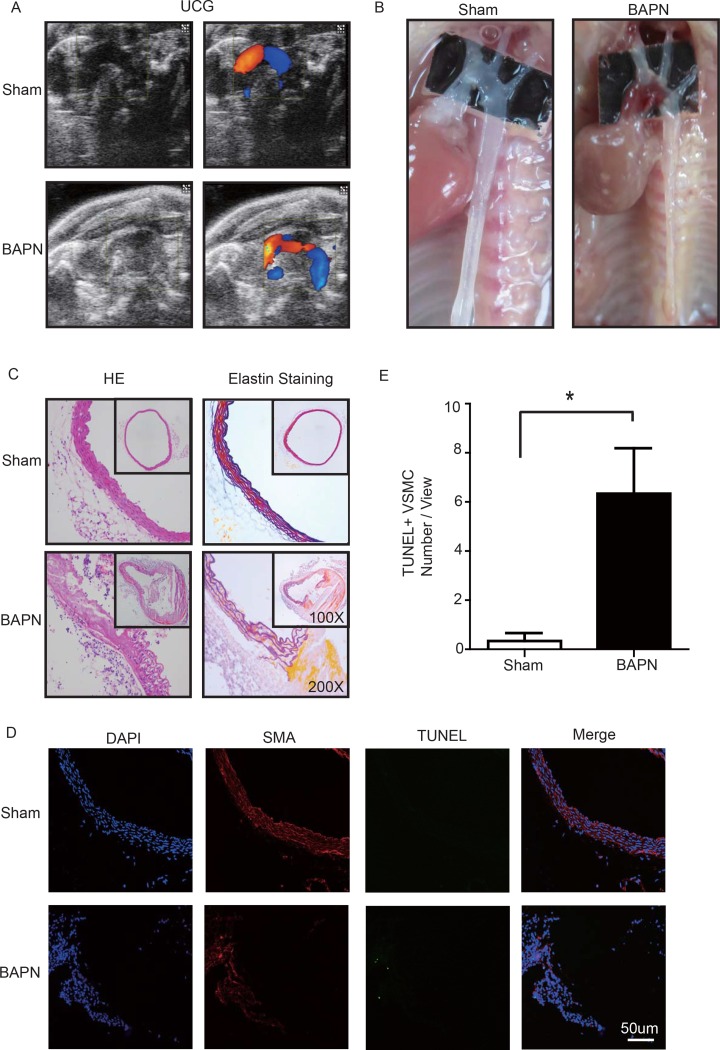
(**A**) Echocardiographic results showing that the area of the ascending aorta without colorful blood flow in the BAPN model suggesting a false cavity of dissection. (**B**) Gross examination revealing mural hematomas in the BAPN model, suggesting a false cavity of dissection. (**C**) H&E and elastin staining showing obvious disorganization of the aortic tissue structure in the BAPN model. (**D, E**) Confocal fluorescence microscopy showing that the cell number of double stained (TUNEL and α-SMA) cells was significantly more in the BAPN-induced ascending aortic dissection model (n=10 in Sham group, n=10 in BAPN group, *p=0.0335).

Lysyl oxidase (LOX), which crosslinks tropoelastin monomers to form elastic fibers, reportedly has a significant role in regulating ECM homeostasis [26]. Thus, BAPN (β-aminopropionitrile monofumarate, Sigma–Aldrich, St. Louis, MO), a LOX inhibitor, was used to build the model of aortic dissection in mice [4, 27]. Through echocardiography and anatomical obser-vation, we evaluated the ascending aortic dissection induced by BAPN after 4 weeks. After 4 weeks of BAPN treatment, the animal models presented with the features of STAAD, as shown in Figure 3A, B. We performed H&E and elastin staining and found VSMC disorganization and elastic lamella dissection that were similar to those observed in the clinical STAAD samples (Figure 3C). We also detected VSMC apoptosis in the ascending aortic wall of mice with BAPN-induced ascending aortic dissection based on TUNEL and α-SMA staining (Figure 3D, E). These results confirmed that the BAPN-induced mouse STAAD model successfully recapitulated the human phenotype, including ECM damage and VSMC apoptosis.

### YAP was down-regulated in ascending aorta of BAPN-induced mouse STAAD model

**Figure 4:**
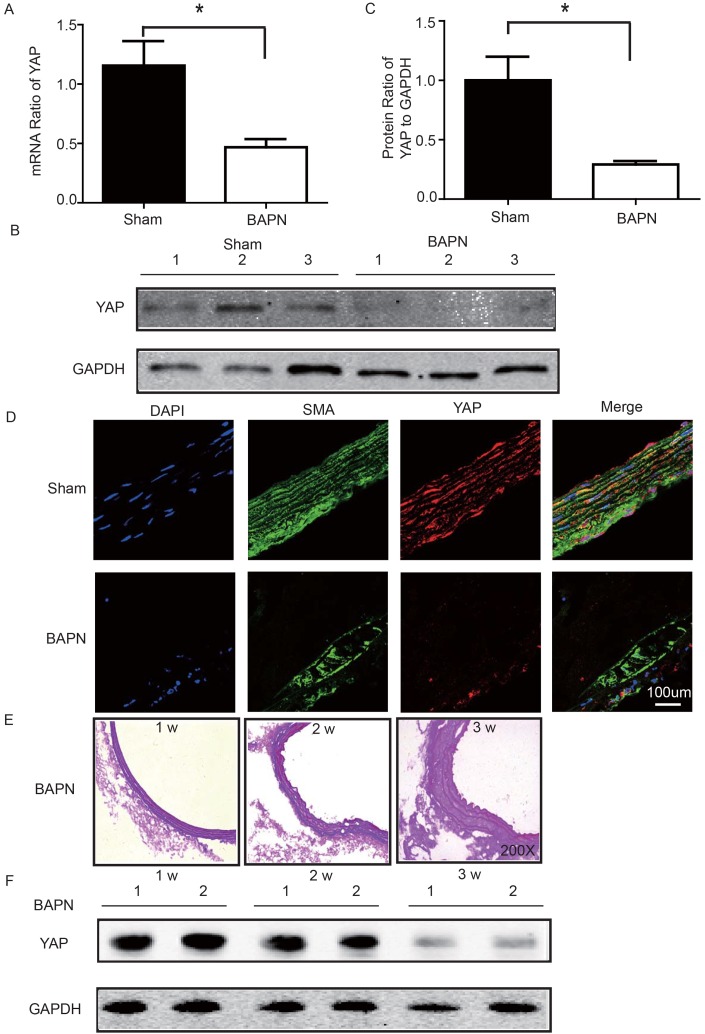
(**A**) Real-time PCR showing that YAP was expressed at significantly lower levels in the ascending aortic wall of the BAPN-induced STAAD mouse compared to that of the sham control (n=10 in Sham group, n=10 in BAPN group, *p=0.0337). (**B, C**) Western blotting showed that the total YAP protein expression was significantly lower in the ascending aortic wall of the BAPN-induced STAAD mouse compared to that of the sham control (n=10 in Sham group, n=10 in BAPN group, *p=0.0088). (**D**) Confocal fluorescence microscopy showing that YAP and α-SMA double stained cells were present at lower number and less expression of YAP in the ascending aortic wall of BAPN-induced STAAD mice. (**E**) Elastin staining of the ascending aortas of mice that were treated with BAPN for different times (1, 2 and 3 weeks) showing that the ascending aortas of mice presented significant elastin disorganization after 3 weeks of BAPN administration compared to mice receiving BAPN for 1 or 2 weeks. (**F**) Western blotting showing that YAP expression in the ascending aorta was lower after feeding with BAPN for 3 weeks than after feeding with BAPN for 2 weeks.

We used the BAPN-induced mouse STAAD model to verify the relationship between down-regulated YAP and STAAD. Consistent with the patient samples, we found that YAP was significantly reduced in the ascending aortic wall of BAPN-induced STAAD mice at both the RNA and protein levels (Figure 4A-C). Confocal fluorescence microscopy analysis of the co-staining of α-SMA and YAP in the tunica media indicated that YAP is mainly expressed in VSMCs in normal aortas; In the BAPN models, the YAP expression level was reduced in the middle layer (Figure 4D). Finally, SMA expression was significantly lower, similarly to the results observed in human STAAD (Figure 4D).

### YAP decreased during development of ECM damage in BAPN-induced mouse STAAD model

To investigate the role of YAP in STAAD development, we examined its temporal expression in the ascending aortas of mice after BAPN administration at various time points (1, 2 and 3 weeks). To assess the extent of ECM damage, we graded elastin degradation (4 grades) [28, 29]. The ascending aortas of mice after BAPN administration presented significant elastin disorganiza-tion at 3 weeks compared to 1 week or 2 weeks (Figure 4E). We then confirmed YAP down-regulation in the ascending aorta over time using western blotting. The observed trends were consistent with the ECM damage (Figure 4F).

### YAP knockdown induced VSMC apoptosis under static conditions

**Figure 5:**
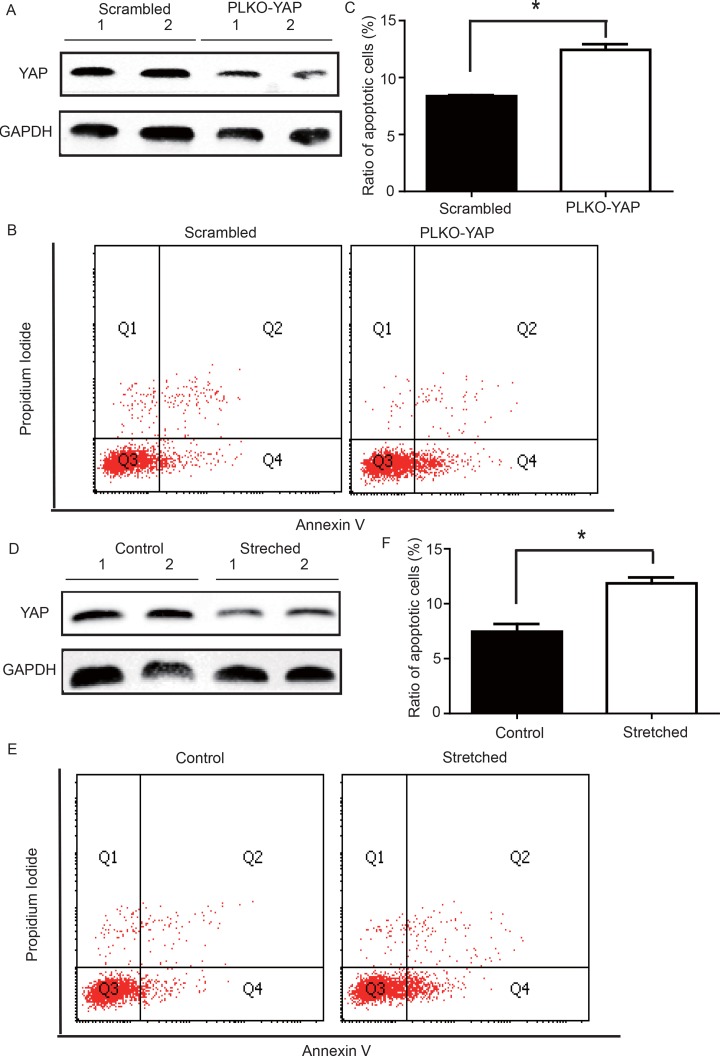
(**A**) Western blot showing YAP down-regulation in the PLKO-YAP group compared to the scrambled group. (**B**, **C**) Flow cytometry showing increased VSMC apoptosis in the PLKO-YAP group compared to the scrambled group (repeated 3 times for statistical analysis, *p=0.0013). (**D**) Western blotting showing YAP down-regulation in the experimental group compared to the control group after cyclic stretching. (**E**, **F**) Flow cytometry showing increased VSMC apoptosis compared to the control group after cyclic stretching *in vitro* (the lower right quadrant (Q4) represents the apoptotic VSMCs, and repeated 3 times for statistical analysis, *p=0.0010).

To determine whether YAP down-regulation induced VSMC apoptosis, an *in vitro* experiment was performed. CRL-1999 VSMCs were infected with a lentivirus expressing either YAP short hairpin RNAs (shRNA) or a scrambled control. Western blotting analysis showed that YAP was successfully knocked down in the PLKO-YAP group compared to the scrambled control group (Figure 5A). Moreover, flow cytometry showed increased VSMC apoptosis after YAP knockdown *in vitro* (Figure 5B). The mean ratio of apoptotic VSMCs was significantly higher in the PLKO-YAP group (12.43±0.50%) compared to the scrambled group (8.37±0.09%, *p*=0.0013, Figure 5C).

### Altered mechanical stress induced YAP down-regulation and apoptosis in cultured VSMC

To determine the possible relationship between the stress-induced YAP down-regulation and VSMC apoptosis, a mechanical strain unit was used to simulate altered mechanical stress in the damaged ECM. CRL-1999 VSMCs in the experimental group were subjected to cyclic mechanical stretching using a computer-controlled mechanical strain unit (18% elongation for 36 hours); CRL-1999 VSMCs in the control group were cultured under normal conditions. After cyclic stretch-ing, western blotting analysis showed that YAP was down-regulated in the experimental group compared to the control (Figure 5D). In addition, flow cytometry showed increased VSMC apoptosis after cyclic stretching *in vitro* (Figure 5E). The mean percentage of apoptotic VSMCs in the experimental group (11.86±0.53%) was significantly larger than that in the control group (7.46±0.49%, *p*=0.0010, Figure 5F).

## DISCUSSION

**Figure 6:**
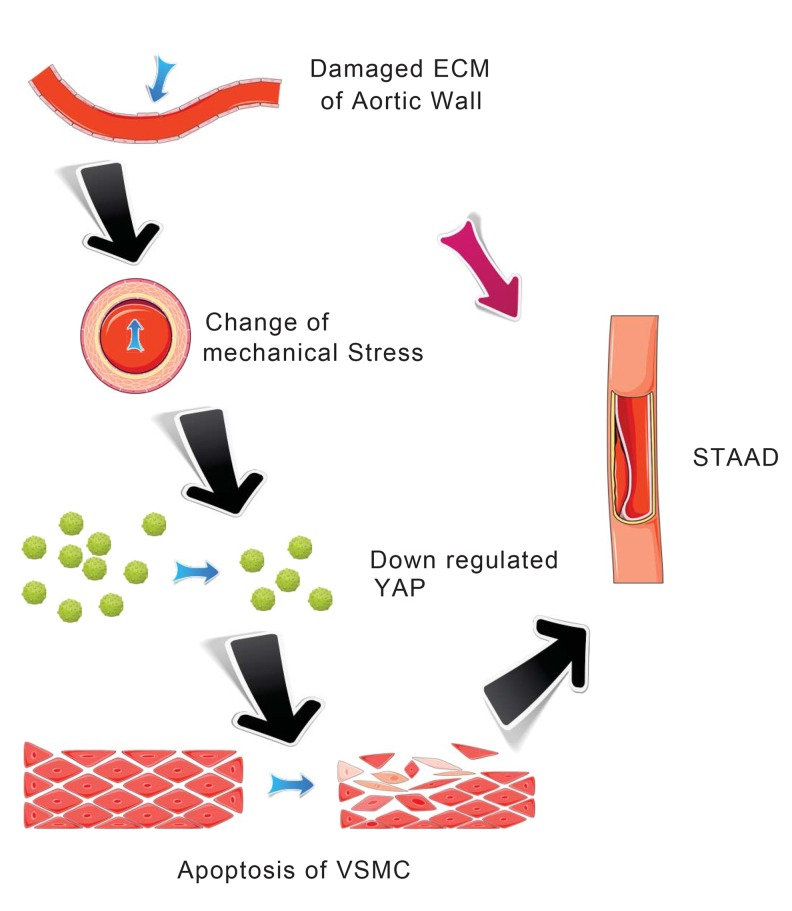
Schematic model showing that the disruption of mechanical stress in ECM induces STAAD and is related to the down-regulation of YAP.

Our main finding was the observation of a unique mechanism of altered mechanical stress caused by the damaged ECM. This altered stress induced YAP down-regulation and VSMC apoptosis, which is related to STAAD development (Figure 6). The elastic properties of the vascular system might represent a true measure of aortic wall weakness. In the clinic, these properties are evaluated based on the stiffness index of aorta, which is negatively related to Vmax (including aortic wall systolic, late diastolic retraction and early diastolic retraction velocities) [5, 30]. Decreased Vmax is predictive of aortic dissection [5, 30]. In this study, the Vmax of the ascending aorta was significantly decreased in patients with STAAD, suggesting that pre-existing weakness represents the histopathological basis of STAAD formation.

VSMC plays a key role in maintaining aortic wall function and substantially contribute to the elastic lamellar architecture of the arterial wall, both directly and indirectly through their production of matrix proteins [31, 32]. Several studies have found that apoptosis was related to the pathology of aortic medial degeneration and in the pathogenesis of aortic aneurysm and dissection [4, 33, 34]. Using TEM, we observed a distinct VSMC apoptotic ultrastructure in the ascending aortic wall of patients with STAAD. Some previous studies have also concluded that the α-SMA-positive cells in the middle layer of the aorta are mainly VSMCs [4, 35, 36]. We found that the α-SMA-positive cells, which were primarily VSMCs, showed an increased intracellular frequency of characteristic TUNEL apoptosis and increased expression of cleaved caspase-3, which were key markers of apoptosis, in STAAD patients’ ascending aortic wall. These results were similar to those found using TEM. Thus, VSMC apoptosis was confirmed to be accompanied by ECM damage. ECM damage can lead to reduced aortic wall tensile strength and elevated vascular wall mechanical stress [4]. In addition, VSMC apoptosis might be induced by cyclic stretching, similarly to the changes induced by mechanical stress [4]. This was also supported by the results found in our mouse BAPN model, in which the failure of elastin crosslinking in the ascending aorta and VSMC apoptosis following exacerbation of ECM damage were observed.

The functional role of YAP has been identified in cardiomyocytes [22] and VSMCs [37]. During cardiovascular development, YAP affects cardiac/SMC proliferation by regulating the expression of some cell cycle suppressors, such as G-protein–coupled receptor 132 (Gpr132) [23]. YAP is also a central regulator of the phenotypic switch of VSMCs [37]. Our previous study found that YAP also plays a key role in hypertrophic cardiomyopathy [22]. Thus, the regulation of cardiovascular development by YAP cannot be overemphasized. YAP is inhibited by culturing cancer cells, such as HeLa cells, on a soft matrix [38]. However, whether a similar phenomenon is conserved in VSMCs is still unknown. In the present study, the results of clinical samples showed that clearly disrupted elastic lamellae of variable widths in STAAD softened the ECM of the ascending aortic wall, possibly inducing YAP down-regulation.

Although we observed an association between ECM damage and YAP expression in STAAD patients’ samples, we were still unable to estimate whether changes in mechanical stress caused the YAP down-regulation. To solve this problem, we developed a mouse BAPN model in which the ECM was disturbed [26]; these mice were prone to aortic dissection [4, 27]. In the present study, ECM damage occurred as early as 2 weeks after BAPN treatment, though decreases in YAP expression did not appear until 3 weeks after BAPN treatment. Gradual decreases in YAP expression were observed following ECM damage in the ascending aortas of the BAPN-treated mice; this finding supports the hypothesis that altered mechanical stress induces YAP down-regulation.

Some researchers have reported that YAP down-regulation can induce apoptosis in cells such as podocytes and PC-3 cells [39, 40]. However, there are no studies showing whether YAP down-regulation induces VSMC apoptosis. To explore this issue, an *in vitro* experiment was used. After YAP knockdown, VSMC apoptosis increased significantly, demonstrating that YAP knockdown under static conditions causes VSMC apoptosis. Considering the complexity of the *in vivo* situation, cyclic stretching has been used to establish an *in vitro* apoptosis model in VSMC [41]. To test the direct effects of mechanical stress on VSMCs, we employed CRL-1999 VSMCs. Changed mechanical stretching induced YAP down-regulation in VSMCs. This suggested that cyclic stretching, which mimics altered mechanical stress in the ascending aorta, led to decreased YAP expression in the cyclic stretch-treated CRL-1999 VSMCs. These results demonstrated that the changes in mechanical stretching led to YAP down-regulation and VSMC apoptosis. Several researchers have reported that knockdown of YAP enhanced contra-ctile phenotype-specific gene expression in VSMCs, including myocardin, SMA, SM22, and SMMHC [37, 42]. Usually, an enhanced smooth muscle contractile phenotype would be beneficial to the function of the aorta. However, given the crucial role of YAP in the proliferation of VSMCs, knockdown of YAP in VSMCs after artery injury instead attenuated the injury-induced conversion of the smooth muscle phenotype [42]. Our present data confirmed this conclusion from a different perspective: knockdown of YAP abolished smooth muscle contractile phenotype-specific gene expression in VSMCs by inducing the apoptosis of VSMCs (Figure 2D), ultimately impairing the function of the aorta and promoting the development of STAAD.

The limitation of this study, the role of major targets of YAP in the pathogenesis of ECM mechanical stress-induced STAAD, will be examined in our future research. In fact, many YAP targets, including transcriptional coactivator with PDZ-binding motif (TAZ)[43, 44], TEAD family transcription factors[44], and the myocardin-SRF complex[37], have been studied in the context of other arterial diseases. All the studies noted above focused on the role of YAP targets in the proliferation of VSMCs: inhibition of TAZ and TEAD reduced VSMCs proliferation [43, 44], and YAP promoted the conversion of VSMCs from a proliferative phenotype to a contractile phenotype by interacting with the myocardin-SRF complex [37]. Additionally, the major YAP targets influencing the apoptosis of VSMCs should be investigated, especially with respect to their roles in ECM mechanical stress-induced STAAD pathogenesis.

In a conclusion, this study reported for the first time that altered YAP expression is associated with ECM damage in STAAD. YAP down-regulation caused by the dis-ruption of mechanical stress is related to the development of STAAD through its induction of aortic VSMC apoptosis.

## MATERIALS AND METHODS

### Study 1

#### Clinical individual collection

The Ethics Committee of Beijing Anzhen Hospital approved the research involving human aortic tissue samples, and all experiments involving human aortic tissue samples were performed in accordance with the guidelines approved by the committee. Informed consent was obtained from all patients. Human normal aortic samples were obtained from 19 HTDs. Human STAAD samples were obtained from 23 patients undergoing surgical replacement of the ascending aorta who suffered from STAAD; the mean age of the participants was 49.6±8.9 years. All patients included presented with acute STAAD, and the mean time period between the diagnosis of STAAD and surgery was 12.8±7.6 hours (Table 1). The mean diameter of the ascending aorta was 47.7±6.8 mm. None of the included patients had bicuspid aortic valve disease. The clinical information obtained for all patients is shown in Table 1. Patients with connective tissue disorders, such as Marfan syndrome was excluded.

**Table 1:** Stanford Type A aortic dissection patient information regarding the aortic tissue used in the study

Patient	Age	Gender	Ascending aorta diameter (mm)	Aortic insufficiency degree	Bicuspid aortic valve disease	Time period between diagnosis of STAAD and surgery (h)	Surgery
1	59	Male	43	Mild	None	25	Ascending aortic replacement+ total arch replacement and frozen elephant trunk implantation
2	44	Male	40	None	None	1	Ascending aortic replacement+ total arch replacement and frozen elephant trunk implantation
3	51	Male	41	Mild	None	2	Ascending aortic replacement+ total arch replacement and frozen elephant trunk implantation
4	60	Female	44	Mild	None	11	Ascending aortic replacement+ total arch replacement and frozen elephant trunk implantation
5	50	Female	35	None	None	15	Ascending aortic replacement+ total arch replacement and frozen elephant trunk implantation
6	61	Male	43	Mild	None	17	Ascending aortic replacement+ patrtial arch replacement+ noncoronary sinus plasty
7	51	Male	46	Mild	None	14	Ascending aortic replacement+ total arch replacement and frozen elephant trunk implantation
8	43	Male	52	Severe	None	21	Bentall+ total arch replacement by a tetrafurcate graft and stented elephant trunk implantation
9	46	Male	40	Severe	None	23	Bentall+ total arch replacement by a tetrafurcate graft and stented elephant trunk implantation
10	41	Female	45	Mild	None	12	Ascending aortic replacement+ total arch replacement and frozen elephant trunk implantation
11	51	Male	45	Severe	None	6	Bentall+ total arch replacement by a tetrafurcate graft and stented elephant trunk implantation
12	55	Male	53	Moderate	None	28	Bentall+ total arch replacement by a tetrafurcate graft and stented elephant trunk implantation
13	50	Male	50	Severe	None	13	Bentall+ total arch replacement by a tetrafurcate graft and stented elephant trunk implantation
14	68	Male	60	None	None	3	Ascending aortic replacement+ total arch replacement and frozen elephant trunk implantation
15	38	Male	52	Severe	None	11	Bentall
16	45	Male	47	Mild	None	19	Ascending aortic replacement+ total arch replacement and frozen elephant trunk implantation
17	37	Male	56	Severe	None	10	Bentall+ total arch replacement by a tetrafurcate graft and stented elephant trunk implantation
18	67	Female	53	Mild	None	16	Ascending aortic replacement+ total arch replacement and frozen elephant trunk implantation
19	36	Male	64	None	None	18	Ascending aortic replacement+ total arch replacement and frozen elephant trunk implantation
20	43	Male	42	Moderate	None	3	Bentall+ total arch replacement by a tetrafurcate graft and stented elephant trunk implantation
21	50	Male	50	Severe	None	12	Bentall+ total arch replacement by a tetrafurcate graft and stented elephant trunk implantation
22	51	Male	48	Severe	None	6	Bentall+ total arch replacement by a tetrafurcate graft and stented elephant trunk implantation
23	44	Male	45	Mild	None	9	Ascending aortic replacement+ total arch replacement and frozen elephant trunk implantation

#### Echocardiography

Patients with STAAD and five age- and gender-matched healthy volunteers underwent transthoracic echocardiography before surgery (GE Corporation). More than half of the included patients had no or mild aortic insufficiency (Table 1). These patients underwent ascending aorta replacement surgery rather than the Bentall procedure. Given the impact of aortic insufficiency, only those patients with no or mild aortic insufficiency underwent echocardiographic velocity assessment. Images were obtained at similar positions in the anterior walls of all ascending aortas, which were also known as the lesser curvature. M-mode diameter measurements were made during systole (the point of maximal anterior motion of the aorta) and at end-diastole (the Q wave on the electrocardiogram) by the same echo-cardiologist. By marking a region of interest on the 2-dimensional image of the anterior aspect of the aorta, velocities were determined throughout the cardiac cycle for this area. The velocity data sets were analyzed off-line using dedicated software (GE Corporation). The means of 5 velocity measurements in sequential cardiac cycles were used in the data analysis.

#### Aorta sample collection

Samples were collected from the same position of the ascending aorta (the anterior walls of all ascending aortas, which were also known as the lesser curvature) in normal individuals and in patients with STAAD. After resection of the ascending aortas during surgery, the tissues were preserved immediately in liquid nitrogen, 4% paraformaldehyde, and 10% formalin.

### Study 2

#### Establishment of a mouse model of ascending aortic dissection

The ethics committee of Beijing Anzhen Hospital approved the research in mice. All animal experiments were performed in accordance with the guidelines approved by the Ethics Committee of Beijing Anzhen Hospital. Three-week-old male mice (C57BL/6) were purchased from Vital River Laboratory Animal Technology Co. Ltd. (Beijing, China). Two animals were housed per cage, maintained in constant temperature-controlled rooms (22-25°C) with a 12-hour light/dark cycle, and given free access to food and water. After acclimatization to the housing conditions, the mice were randomly assigned to two groups, one group received 1 g/kg per day of BAPN, which was dissolved in the drinking water (BAPN group) and one group received solvent (sham group). The BAPN dose was adjusted according to body weight, which was monitored weekly. At the end of the treatment (for 1, 2, 3 and 4 weeks), the mice were observed as previously described [45].

#### Aortic ultrasonographic monitoring

Color Doppler echography in the M-mode using a high-resolution micro-ultrasound system (Vevo 2100, VisualSonics) equipped with a 30-MHz transducer was used to detect arterial flow after the mice were anaesthetized using 1% isoflurane. Confirmation was provided by an operator who was blinded to the animal group.

### Study 3

#### Cell culture

CRL-1999 human aortic VSMCs were cultured at 37°C in ATCC-formulated F-12K Medium (Catalog No. 30-2004). To prepare the complete growth medium, the following components were added to the base medium (final concentrations): 0.05 mg/ml ascorbic acid; 0.01 mg/ml insulin; 0.01 mg/ml transferrin; 10 ng/ml sodium selenite; 0.03 mg/ml endothelial cell growth supplement (ECGS); 10% fetal bovine serum; 10 mM HEPES; and 10 mM TES. 293T cells were cultured at 37°C in 5% CO_2_ Dulbecco's modified Eagle's medium (Invitrogen) supplemented with 10% fetal bovine serum (Invitrogen) and 100 U/ml penicillin and streptomycin (Gibco).

#### Infection with shRNA lentivirus

YAP knockdown was achieved by infection with a lentivirus expressing either YAP shRNA or scrambled control. All of the shRNAs were cloned into plko.1-puro vectors. (a generous gift from Dr. Zengqiang Yuan, Institute of Biophysics, Chinese Academy of Sciences, Beijing, China) [46]. Cultured CRL-1999 VSMCs were infected with these viruses and then screened using 2, 6 or 10 μg/ml kitazine. The cells that survived in 10 μg/ml kitazine were used in a follow-up study. For simplicity, the resulting cells will be referred to as “scrambled” VSMCs and “PLKO-YAP” VSMCs throughout this work. This cell culture experiments were repeated 3 times for statistical analysis.

#### Cyclic stretch

Before the cyclic stretch procedure, CRL-1999 VSMCs were cultured on silicone elastomer-bottomed collagen-coated plates with 70% seeding density (Flexcell Inc.) at 37°C overnight. CRL-1999 VSMCs were treated as described [4] with a cyclic mechanical stretch uniaxially, which was applied using a computer controlled mechanical strain unit (Flexcell 5000) set to 18% elongation (stretched group). The frequency, at which the VSMCs were stretched, was 60 stretches per minute to simulate a human normal heart rate. This cell culture experiments were repeated 3 times for statistical analysis.

### General techniques

#### Transmission electron microscopy

Briefly, tissues were fixed in buffered 3% glutaral-dehyde for 2 hours, washed in 0.1 mol/L phosphate buffer solution, and post-fixed using 1% osmium tetroxide. The samples were then washed in 0.1 mol/L phosphate buffer solution, dehydrated in a graded ethanol series, and embedded in Epon 812. Thick sections (approximately 0.5 μm) were stained with toluidine blue, and the sections were used to determine the lobular orientation of each tissue block. Thin sections were cut from the tissue block using an LKB-V ultramicrotome (LKB, Bromma) and then doubly stained using uranyl acetate and lead citrate. The ultrastructural features were observed and photographed using an H-800 transmission electron microscope (TEM, Hitachi).

#### Aorta elastin staining

Human and mouse aortas were stained for elastin using an elastic fiber staining kit following an established method (Maixin Bio., Fuzhou, China) [4]. Paraffin was removed by treating the sections with xylene, followed by rehydration. Four consecutive 5-μm sections were collected on each slide, and ten slides were prepared for each sample. The sections were then stained by incubation for 5 min in Lugol's iodine solution, washing with phosphate-buffered saline (PBS), incubation with sodium thiosulfate for 5 min, washing with PBS and 70% ethanol, incubation with aldehyde-fuchsine for 10 min, and a final incubation step with acid Orange G for several seconds.

#### Histological staining (H&E) and immunohisto-chemistry

Aortic samples were fixed in 10% formalin and embedded in paraffin. Four consecutive 5-μm sections were collected on each slide. Ten slides were prepared for each sample. The sections were stained using established methods [4]. Paraffin was removed by treating the sections with xylene, followed by rehydration. The sections were incubated in 3% H_2_O_2_ for 10 min at room temperature and then washed 3 times with PBS. After 30 min of blocking, the sections were incubated with primary antibodies against YAP (1:1000 dilution, Cell Signaling), α- smooth muscle actin (α-SMA, 1:500 dilution, Sigma), or cleaved-caspase 3 (1:300 dilution, Cell Signaling). Controls for immuno-specificity were included in all experiments, and the primary antibody was replaced by a matching normal IgG. The antibody-stained sections were then incubated using the ChemMate^TM^ EnVision^TM^ System (Dako, Glostrup). Image ProPlus 3.0 (ECIPSE80i/90i) was used to capture the images and analyze the results. Quantitative analysis of immunohistochemical staining was performed by two experienced operators who were blinded to the study protocol. Five unfolded continuous fields in each section were examined.

#### Immunofluorescence

For cryostat sections, aortic samples were fixed in 4% paraformaldehyde, embedded in optimum cutting temperature (OCT) compound, and frozen in liquid nitrogen Four consecutive 5-μm sections were collected on each slide, and ten slides were prepared for each sample. A TUNEL assay (Promega) was used to detect apoptotic cells. An anti-α-SMA antibody was used in the same section, which was then incubated with the corresponding secondary antibodies. The slides were then assessed using confocal fluorescence microscopy analysis (Leica Microsystems).

#### Western blot analysis

Human and mouse aortic samples or cultured VSMCs were harvested. Proteins were extracted using a protein extraction kit containing protease inhibitor and protein phosphatase inhibitor cocktail. Equal amounts of protein extracts (40 μg/lane) were resolved using 10% SDS-PAGE. Target protein expression was probed using anti-YAP (1:1,000 dilution, Cell Signaling) as the primary antibody for over 6 hours at 4°C. The samples were then probed using IR dye-conjugated secondary antibodies (1:5,000, Rochland Immunochemicals, Gilbertsville, PA) for 1 hour. The same membrane was blotted using anti-GAPDH (glyceraldehyde-3-phosphate dehydrogenase, 1:2,000 dilution, Sigma-Aldrich). The results were analyzed using an Odyssey infrared imaging system (LI-COR Biosciences Lincoln, NE).

#### RNA isolation and real-time PCR assay

Total RNA was isolated from aortic samples using Trizol reagent (Invitrogen), and cDNA was synthesized using 1 μg of total RNA and random hexamer primers (Takara) in a 20-μl volume using the GoScript™ Reverse Transcription System (Promega). Specific genes were amplified using real-time quantitative PCR, in which 1 μl of the reverse transcription product was amplified using the iQ5 system (Bio-Rad, Hercules, CA) and SYBR Green I (Takara). The relative abundance of each mRNA was determined after normalization to GAPDH mRNA. The primers used are listed in Supplementary Table S1.

#### Flow cytometry

The apoptosis protein Annexin V was detected using a kit (eBioscience) following the manufacturers’ protocol, and the number of apoptotic CRL-1999 VSMCs was determined using flow cytometry. According to the previous studies [47, 48], the lower right quadrant (Q4) represents the apoptotic VSMCs (Annexin V positive and PI negative), while the upper right quadrant (Q2) contains the nonviable necrotic VSMCs (positive for Annexin V binding and for PI uptake). So the level of VSMC apoptosis was reported based on Q4 in the present study.

#### Statistical analysis

The data were analyzed using a two-tailed Student's *t*-test through Prism 5.01 for Windows XP. The data are presented as the means ± SEM (except in the luciferase assay data, where the means ± SD are presented).* *p* <0.05 was considered significant.

## SUPPLEMENTARY MATERIAL TABLE


