# Supplementary material for: Disruption of mechanical stress in extracellular matrix is related to Stanford type A aortic dissection through down-regulation of Yes-associated protein
Source: Aging (Albany NY). 2016 Sep 5;8(9):1923–38. doi: 10.18632/aging.101033 (PMC5076445; doi:10.18632/aging.101033)
Supplement: Supplementary file 1 [file aging-08-1923-s001.pdf]

SUPPLEMENTARY MATERIAL

Supplementary Table 1. Primers Used for Real-time PCR

Gene	Primer Sequence	PrimerBank ID
Human Yap-5f	Forward: 5' - TAGCCCTGCGTAGCCAGTTA - 3'	303523510c1
Human Yap-3r	Reverse: 5' - TCATGCTTAGTCCACTGTCTGT - 3'	
Human Gapdh-5f	Forward: 5' - GGAGCGAGATCCCTCCAAAAT - 3'	378404907c1
Human Gapdh -3r	Reverse: 5' - GGCTGTTGTCATACTTCTCATGG - 3'	
Mouse Yap-5f	Forward: 5' - ACCCTCGTTTTGCCATGAAC - 3'	15928514a1
Mouse Yap-3r	Reverse: 5' - TGTGCTGGGATTGATATTCCGTA - 3'	
Mouse Gapdh-5f	Forward: 5' - AGGTCGGTGTGAACGGATTTG - 3'	126012538c1
Mouse Gapdh-3r	Reverse: 5' - GGGGTCGTTGATGGCAACA - 3'	
